# Correction: “It’s wishy-washy [...] You are getting this diagnosis because we’ve ruled out everything else.” Developmental language disorder (DLD) diagnosis in the Republic of Ireland: A qualitative exploration of the perspectives of parents and clinicians

**DOI:** 10.1371/journal.pone.0335868

**Published:** 2025-10-31

**Authors:** The PLOS One Staff

## Notice of republication

This article was republished on October 20, 2025, to remove a colon from the title. The correct title is “It’s wishy-washy [...] You are getting this diagnosis because we’ve ruled out everything else.” Developmental language disorder (DLD) diagnosis in the Republic of Ireland: A qualitative exploration of the perspectives of parents and clinicians. Please download this article again to view the correct version. The originally published, uncorrected article and the republished, corrected articles are provided here for reference.

## Supporting information

S1 File Originally published, uncorrected article. (PDF)

S2 File Republished, corrected article. (PDF)
