# Supplementary material for: “It’s wishy-washy [...] You are getting this diagnosis because we’ve ruled out everything else.” Developmental language disorder (DLD) diagnosis in the Republic of Ireland: A qualitative exploration of the perspectives of parents and clinicians
Source: PLoS One. 2025 Jul 11;20(7):e0327373. doi: 10.1371/journal.pone.0327373 (PMC12250196; doi:10.1371/journal.pone.0327373)
Supplement: S1 Appendix — (DOCX) [file pone.0327373.s001.docx]

**Informed Consent**

**Research Study Title**

Exploring the practices and perspectives of receiving a Developmental Language Disorder (DLD) diagnosis in Ireland: The views of clinicians and parents.

**Principal Investigator**: Dr Sylwia Kazmierczak-Murray

**Contact Email**: sylwia.kazmierczakmurray@dcu.ie

**Co-Investigators**: Dr Neil Kenny, Suzanne Carolan

School of Inclusive and Special Education, Institute of Education, Dublin City University

**Clarification of the purpose of the research**

This project aims to explore whether and in what way SLTs communicate DLD diagnosis, and what are their specific practices and approaches in this area, including if and how they include the families in decision-making. We seek your permission to participate in this study to explore your perspectives and experiences of this topic.

**Confirmation of the researcher’s role and actions:**

The researcher’s remit is to engage participants in a discussion during focus group sessions focused only on the agreed interview questions/schedules. The researchers will listen actively, use audio recording and notation to gather the opinions and perspectives of participants. At all times the researchers will comply with legal guidelines and the ethical standards as set out in the ethical approval for this project.

**Confirmation of participant’s engagement during the project: What will they be doing?**

Participants will voluntarily attend an online focus group and will be given opportunities to discuss their experience of DLD as a ‘diagnostic label’. They will answer questions they feel comfortable answering and are not obliged to answer all questions. Participants may opt out of the focus group at any time. All focus group participant identities will be pseudonymised.

**Please circle the appropriate consent statements**

*We*/*I have read the Plain Language Statement (or had it read to me)                       Yes/No*

*We*/*I understand the information provided                                                                     Yes/No*

*We/I have had an opportunity to ask questions and discuss this study                      Yes/No*

*We/I have received satisfactory answers to all my questions                                Yes/No*

*We are/I consent to the interview being audio recorded                                                       Yes/No*

*We/I understand that involvement in this research project is voluntary                          Yes/No*

(participants my withdraw at any point)

*We/I understand that all data collected will be anonymised and subsequently stored for 2 years by the Principal Investigator. The data will then be destroyed and* *permanently deleted by the PI.* *All data collected will be kept confidential.* *Participant data will be stored in a password protected Google Drive folder.* *Audio-recordings of interviews will be destroyed once they have been transcribed.*

*Yes/No*

*We/I consent to the use of my/our data for future studies and publications as outlined             Yes/No*

*We/I understand that the confidentiality of information provided is subject                                to legal limitations, in accordance with the guidelines of Dublin City University.*

*Yes/No*

Signature:

I have read and understood the information in this form. My questions and concerns have been answered by the researchers, and I have a copy of this consent form.  Therefore, I consent to take part in this research project

Participants Signature:

Name in Block Capitals:

**Research Ethical Approval**

Ethical approval for this research study has been obtained from the Dublin City University Research Ethics Committee.

 If you require more information, please feel free to contact me at the email address below.

Yours sincerely,

Sylwia Kazmierczak-Murray

[Sylwia.kazmierczakmurray@dcu.ie](mailto:Sylwia.kazmierczakmurray@dcu.ie)

**If participants have concerns about this study and wish to contact an independent person,** **please contact:**

The Secretary, Dublin City University Research Ethics Committee, c/o Research and Innovation Support, Dublin City University, Dublin 9.  Tel 01-7008000.
