# Supplementary material for: “It’s wishy-washy [...] You are getting this diagnosis because we’ve ruled out everything else.” Developmental language disorder (DLD) diagnosis in the Republic of Ireland: A qualitative exploration of the perspectives of parents and clinicians
Source: PLoS One. 2025 Jul 11;20(7):e0327373. doi: 10.1371/journal.pone.0327373 (PMC12250196; doi:10.1371/journal.pone.0327373)
Supplement: S2 Appendix — (DOCX) [file pone.0327373.s002.docx]

**Focus Group Schedule: IASLT Members (SLTs)**

1. What are your general views on the DLD as a distinct diagnostic category/label?
2. How does this ‘diagnostic label’ influence support pathways for children and young people in Ireland?
3. What influences you to make a decision about whether or not to ‘issue’ the diagnosis of DLD?
4. Do you engage with parents to make decisions about whether or not to ‘issue’ the diagnosis of DLD?
5. Do you engage with children and young people to make decisions about whether or not to ‘issue’ the diagnosis of DLD?
6. What are the specific practices and approaches that you use in your practice to communicate this ‘diagnostic label’ to children and young people?
7. What are the challenges in communicating this ‘diagnostic label’ to children and families?

**Focus Group Schedule: Parents**

1. What is your experience of how DLD diagnosis was ‘given’ and ‘communicated’ to your child?
2. What was the impact of the DLD diagnosis on your child?
3. What are your views on the ‘timing’ of this diagnosis in your child’s life?
4. What is your experience of how DLD as a ‘diagnostic label’ was ‘communicated’ to you?
5. How was this diagnosis communicated to your child?
6. What are your recommendations regarding the communication of a diagnosis of DLD to parents and children?
