# Supplementary material for: Correction: “It’s wishy-washy [...] You are getting this diagnosis because we’ve ruled out everything else.” Developmental language disorder (DLD) diagnosis in the Republic of Ireland: A qualitative exploration of the perspectives of parents and clinicians
Source: PLoS One. 2025 Oct 31;20(10):e0335868. doi: 10.1371/journal.pone.0335868 (PMC12578134; doi:10.1371/journal.pone.0335868)
Supplement: S1 File — (PDF) [file pone.0335868.s001.pdf]

PLOS ONE

CORRECTION

Correction: Association of dietary sodium intake with impaired fasting glucose in adult cancer survivors: A population-based cross-sectional study

Kyuwoong Kim, Haemee Kim, Tae Jun, Young-Hak Kim

The second author's name is spelled incorrectly. The correct name is: Haemee Kim.

In the Funding statement, the grant number from the funder Ministry of Health and Welfare, Republic of Korea is listed incorrectly. The correct grant number is: HR21C0198.

Reference

1. Kim K, Kim H, Jun TJ, Kim Y-H (2023) Association of dietary sodium intake with impaired fasting glucose in adult cancer survivors: A population-based cross-sectional study. PLOS ONE 18(5): e0286346. https://doi.org/10.1371/journal.pone.0286346 PMID: 37228155

Check for updates

OPEN ACCESS

Citation: Kim K, Kim H, Jun T, Kim Y-H (2024) Correction: Association of dietary sodium intake with impaired fasting glucose in adult cancer survivors: A population-based cross-sectional study. PLoS ONE 19(11): e0314645. https://doi.org/10.1371/journal.pone.0314645

Published: November 21, 2024

Copyright: © 2024 Kim et al. This is an open access article distributed under the terms of the Creative Commons Attribution License, which permits unrestricted use, distribution, and reproduction in any medium, provided the original author and source are credited.
