# Supplementary material for: Correction: “It’s wishy-washy [...] You are getting this diagnosis because we’ve ruled out everything else.” Developmental language disorder (DLD) diagnosis in the Republic of Ireland: A qualitative exploration of the perspectives of parents and clinicians
Source: PLoS One. 2025 Oct 31;20(10):e0335868. doi: 10.1371/journal.pone.0335868 (PMC12578134; doi:10.1371/journal.pone.0335868)
Supplement: S2 File — (PDF) [file pone.0335868.s002.pdf]

PLOS One

RESEARCH ARTICLE

"It's wishy-washy [...] You are getting this diagnosis because we've ruled out everything else." Developmental language disorder (DLD) diagnosis in the Republic of Ireland: A qualitative exploration of the perspectives of parents and clinicians

Sylwia Kazmierczak-Murray[1*], **Neil Kenny**[1], **Suzanne Carolan**[2], **Alison Doyle**[3]

1 Institute of Education, Dublin City University, Ireland, **2** Louth Primary Care, Health Service Executive, Ireland, **3** Caerus Education, Ireland

* sylwia.kazmierczakmurray@dcu.ie

Check for updates

OPEN ACCESS

Citation: Kazmierczak-Murray S, Kenny N, Carolan S, Doyle A (2025) "It's wishy-washy [...] You are getting this diagnosis because we've ruled out everything else." Developmental language disorder (DLD) diagnosis in the Republic of Ireland: A qualitative exploration of the perspectives of parents and clinicians. PLoS One 20(7): e0327373. https://doi.org/10.1371/journal.pone.0327373

Editor: Ramandeep Kaur, Father Muller Charitable Institutions, INDIA

Received: May 6, 2025

Accepted: June 13, 2025

Published: July 11, 2025

Copyright: © 2025 Kazmierczak-Murray et al. This is an open access article distributed under the terms of the Creative Commons Attribution License, which permits unrestricted use, distribution, and reproduction in any medium, provided the original author and source are credited.

Data availability statement: The dataset for this study comprises in-depth qualitative interviews focused on the process of DLD

Abstract

Developmental Language Disorder (DLD) affects around 7% of children globally, yet the scholarship on it is significantly underdeveloped. Emerging research suggests that DLD is both underdiagnosed by clinicians and misunderstood by parents. This study explores the perspectives of both clinicians and parents regarding their experiences of the process of giving and receiving a DLD diagnosis in the Republic of Ireland. Semi-structured qualitative group interviews were conducted with 15 parents and seven clinicians. The data were analysed using a reflexive, thematic analysis. Four themes were identified: "challenges of giving a DLD diagnosis", "communicating a DLD diagnosis", "utility of DLD diagnosis for children and families" and "going forward and recommendations". Clinicians reported systemic barriers in the healthcare system, including limited therapy time, long waitlists, and staff turnover, as major challenges in diagnosing DLD. Many expressed a lack of confidence in providing a diagnosis without multidisciplinary support which would support them in 'ruling out' other neurodevelopmental differences. Communicating the diagnosis was often inconsistent, with many parents feeling unsupported and uninformed about the nature and impact of DLD. Parents felt inadequate at being left with communicating the diagnosis of DLD and its impact to their children. The participants emphasised urgent need for greater awareness, teacher and clinical education, post-diagnosis support, and increased national advocacy in relation to DLD in Ireland. Both clinicians and parents saw DLD diagnosis as essential for accessing therapeutic and educational support, yet it was the access to these supports that seemed to influence the diagnostic decisions. Importantly, our research documents clinicians' fear of getting the "right" condition for diagnosis, which may be firstly at odds with the individual profiles

diagnosis. As the transcripts may include potentially identifiable and sensitive health-related information, and given the highly personal nature of the data, full transcripts cannot be publicly shared. This restriction is in accordance with the ethical approval granted for the study, which has approved the study under specific data privacy and confidentiality measures. We confirm that the excerpts of the transcripts relevant to the study are available within the paper. Data access requests may be directed to The Secretary, Dublin City University Research Ethics Committee, c/o Research and Innovation Support, Dublin City University, Ireland at rec@dcu.ie.

Funding: Dublin City University's (DCU) Centre for Inclusive Pedagogy funded the cost of professional transcription and data curation in the study. The funding was acquired by the second author (Neil Kenny). The funder (DCU Centre for Inclusive Pedagogy) had no role in study design, data collection and analysis, decision to publish, or preparation of the manuscript.

Competing interests: The authors have declared that no competing interests exist.

of children, and secondly, acts as a barrier in accessing the needed support. In light of growing awareness of the co-occurrence of neurodevelopmental differences, we call for enhanced support for clinicians to build their confidence in navigating the evolving diagnostic criteria of DLD.

Introduction

Developmental language disorder (DLD) is considered the most prevalent childhood disability, estimated to influence the lives of approximately 7% of children and young people globally [1,2]. Much has been written about its poor awareness among parents, teachers, and the wider society [3] and increasingly, about its negative academic [4] and lifelong impact [5,6]. Despite its prevalence and impact, research scholarship on DLD, albeit growing, remains significantly globally underdeveloped [3,7].

Concerningly, despite its prevalence, research from the last three decades shows that DLD continues to be actually rarely diagnosed [8]. This may be partly attributed to the history of terminological confusion associated with language disorders [9], and previously termed Specific Language Impairment (SLI), the diagnosis of which was viewed by some scholars as ‘unnecessary’ [10] and/or too heterogeneous to have any diagnostic validity [11]. For example, Wright [10] argued that on practical grounds in terms of securing support for children who need it, the exact SLI classification was not needed. This echoes a perspective of Elliot and Grigorenko [12] in relation to dyslexia, who argue that a dyslexia diagnosis adds little value, and further, that its existence may be a major disservice to many children with difficulties learning to read.

Thankfully, since the CATALISE study [1], which proposed new classification to understand childhood speech, language and communications needs, we now should have a clear understanding of DLD and clarity of its diagnostic criteria. Recent research by Gallagher et al. [13], however, has shown that, despite the Irish Association of Speech and Language Therapists (IASLT)’ dissemination efforts to increase Irish clinicians’ knowledge and understanding of the CATALISE recommendations, there exists a lack of professional confidence among clinicians in relation to diagnosing DLD. Gallagher et al. [13] suggested that this may be due to the changed DLD prescriptive criteria which, after the CATALISE consensus [1], are not as “tightly prescribed” and thus require greater professional judgment (i.e., clear cut off scores and a cognitive discrepancy were removed from the prescriptive criteria).

In the context of speech and language discipline, poor and/or inconsistent diagnostic disclosure practices have been documented previously by Tighe & Namazi [8], who found that clinicians are often ineffective at communicating to parents and caregivers *comprehensible* diagnostic terms for their child’s difficulties. Previous studies reported that the parents of children with language difficulties are often not given a clear diagnostic term that would explain their children’s difficulties *at all* [14] or that they do not fully understand it [14,15].

Parents' experience of *receiving* their children's diagnosis has not been sufficiently studied in general, even in relation to neurodevelopmental differences that have been extensively researched (i.e., autism). For example, while there are many studies on autism diagnosis as such, not many of them explore parents' experience of this diagnosis per se [16]. Research is even more limited in relation to communicating diagnostic information to children, and especially as it pertains to children's participation in the context of receiving a diagnosis of a *language* disorder.

Research on the issues and dilemmas around a process of disclosing and communicating clinical labels to families has grown somewhat in recent years, however, mainly addressed in relation to autism [17]. Compared to other neurodevelopmental differences, despite recent advances (e.g., RADLD), Developmental Language Disorder (DLD) continues to receive relatively less attention and advocacy [18]. For example, earlier research in the UK by Roulstone & Lindsay [19] revealed that parents did not use specific labels in relation to the child's communication impairment, despite using such labels in relation to other differences, for example, dyslexia or attention deficit hyperactivity disorder.

Tighe & Namazi [8] proposed that a sensitive and *informative* process of diagnostic disclosure is critical as it serves as a "launching point for an accordant working alliance" between clinicians and families. Consequently, our research started with the following overarching questions:

1) What are the specific practices and approaches used by SLTs in communicating DLD diagnosis to children and families?
2) What are parents' views regarding how the diagnosis of DLD was communicated to them and their child?
3) What are the challenges and supports for clinicians in this area?
4) What are the recommendations or preferences of parents regarding the communication of a diagnosis of DLD to parents and children?

Method

The research was carried out using an exploratory, qualitative design, which is suitable for topics where there is limited available knowledge. The research received ethical approval from the Research Ethics Committee of Dublin City University prior to the start of the study and adhered to the Consolidated Criteria for Reporting Qualitative Studies [COREQ; 20].

Research team positionality

The research team included two female and one male researchers and a female clinician. As a team of researchers, we have a multidisciplinary background (Speech and Language Therapy, Psychology, and Education), and extensive both clinical and research experience. This study was our first collaborative research project.

Participants

Clinicians were recruited via the Irish Association of Speech and Language Therapists (IASLT) social media page and via an email to the members of the IASLT Developmental Language Disorder (DLD) Special Interest Group (SIG). The volunteering participants were all qualified SLTs, registered with CORU (Irish Health and Social Care Council), and they all had at least two years of clinical experience. Two years of clinical experience is considered sufficient to accumulate casework experience in this area and this length of experience was also considered in previous studies on DLD [21]. All SLTs who took part in the study were employed by the Health Sector Executive (HSE) Primary Care which means that they all worked with the general paediatric population and thus had current clinical engagement with children and young people with DLD. All SLTs were female. We did not ask for the age of the participants as we considered this was not an influencing factor, instead the two years of *clinical* experience was sufficient. In addition, all SLTs were members of the

Irish Association of Speech and Language Therapists (IASLT) DLD Special Interest Group (SIG) and as members of this group (which organises annual SIG events focused on DLD) had a good understanding of DLD diagnostic criteria.

Parents were recruited via a DLD Parents Facebook Support Group. Fifteen parents participated, including 13 mothers and two fathers. All had a child with a formal DLD diagnosis and were currently engaged in ongoing therapy, either through the HSE Primary Care system or through attendance in a special class where speech and language therapy is provided as part of the curriculum. The time since diagnosis varied across participants, but all had been engaged with services for at least six months at the time of the study. We did not collect detailed background data (e.g., socioeconomic or educational background) to minimise participant burden and because our focus was on shared themes in parent-professional communication rather than demographic correlations. However, the shared platform for recruitment and the therapy engagement of all participants ensured some consistency in experience.

Overall, we recruited 24 participants. In total, we conducted three individual and two dyad interviews with seven clinicians and five focus groups with 15 parents, in dyads and/or triads; one parent and one clinician did not attend the scheduled focus groups. Each interview or focus group was facilitated by one of the authors and audio-recorded; the first three authors were involved in facilitating focus groups and conducting the interviews. The first and third author are members of the Irish Association of Speech and Language Therapy (IASLT) Developmental Language Disorder (DLD) Special Interest Group (SIG), however, they had no personal or professional relationship with any of the participants who took part in the research. One clinician was known to one researcher and so they were interviewed by a different researcher.

Procedure: interviews and focus groups

The frameworks for both the interviews and focus groups were collaboratively developed by the research team, drawing upon existing literature and clinical expertise to ensure alignment with the study's objectives [22]. The guides underwent informal pilot testing within the team to refine question clarity and flow [23], before being formally piloted by the Chair of the IASLT DLD SIG. All interviews were conducted in English, the first language of all clinician participants and the primary language of most parent participants. Participants with English as an additional language confirmed their comfort in participating without translation services. Given the absence of translation, issues related to back-translation or interpreter involvement were not applicable [24–26].

All interviews and focus groups were held online on an institutionally licensed encrypted Zoom platform. The parents' focus groups lasted between 36 and 63 minutes, and the clinicians' interviews or focus groups lasted between 25 and 37 minutes, depending on the participants' elaboration on the topic and the number of participants (i.e., individual or dyads). Data were collected between August 2024 and November 2024. The focus group schedule consisted of open-ended questions. We developed two sets of questions, one for clinicians and one for parents. The questions were shared with the participants prior to their participation in the interviews or focus group and the discussion was hosted at a time convenient to the participants (i.e., we hosted both morning and evening groups). Field notes were made by the respective researchers who facilitated the interviews and focus groups. The interviews and focus groups were audio recorded and transcribed verbatim by a professional transcription service. Personal data was pseudonymised on transcription.

Data analysis

We adopted a five-stage reflexive thematic analysis approach to guide our analysis [21]; we deemed this approach both theoretically flexible and sufficiently disciplined. This method merged the two principal approaches of a top-down, deductive process utilising a priori codes extracted from the research questions, aims, and objectives, and data collection artefacts, i.e., focus group questions, together with a bottom-up, inductive process extrapolated from close reading of the data in three separate cycles.

Firstly, all transcripts were imported into NVivo for analysis. In Bingham's [27] stage one (i.e., 'organising the data'), an initial codebook was created by the research team with each code allocated a definition, inclusion criteria, and exclusion criteria to facilitate cross-coding comparison amongst the team. The first cycle of data analysis was completed by the fourth author (stage two: 'sorting the data') and utilised this a priori codebook to code data against the principal themes identified from the research aim, objectives and interview questions, and also identified the emerging themes for each group. Once this initial coding was complete, related codes were grouped and organized into broader categories.

In stage three (i.e., 'understanding the data'), after discussion amongst the research team, emerging themes and nuances were added to the codebook together with their concomitant definitions, inclusion and exclusion criteria, resulting in a second, updated codebook. Coding summaries containing illustrative extracts for both groups of participants were subsequently discussed by the research team in terms of identifying the need to merge, collapse, or reject data, and a third and coding structure was agreed – integrating data from both groups – and applied to a third and final pass which resulted in four overarching themes. This represented Bingham's [27] stage four of analysis: 'interpreting the data' and began stage five: 'explaining the data'. At this stage, qualitative transcripts were reviewed by the first two authors to ensure the themes were consistent and represented participant perspectives.

The research team met four times during the data analysis process, for peer debriefing and review of the emerging categories and themes. In January 2025, a summary of themes emerging from the research was presented at the IASLT DLD SIG meeting which was attended by the clinicians who took part in the research; clinicians had the opportunity to provide feedback on the findings at this meeting. Both parents and clinicians were asked for feedback on the key findings by email in April 2025.

Results

Four major themes emerged from the data: (1) challenges of DLD diagnosis, (2) communicating a DLD diagnosis, (3) utility of DLD diagnosis for children and families, and (4) going forward and recommendations. Each theme contains sub-themes that illustrate participants' lived experiences (Fig 1).

1. Challenges of DLD diagnosis

Participants described the diagnosis of Developmental Language Disorder (DLD) as a complex and often extended process, shaped by a combination of practical service constraints and professional uncertainties. Clinicians described DLD not as a single decision point but as a gradual, iterative process requiring extended observation and collaboration with

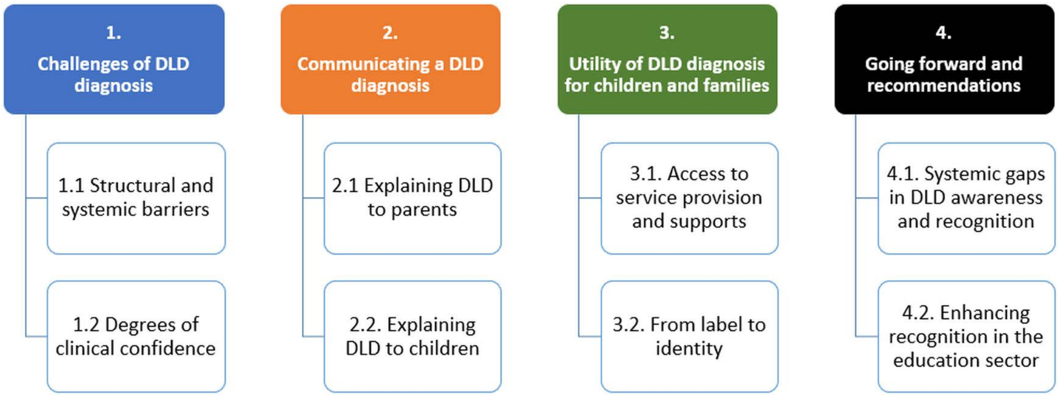

Fig 1. Integrated Themes for Clinician and Parent Groups after Third Coding Pass.

https://doi.org/10.1371/journal.pone.0327373.g001

families, schools, and other professionals. They noted that while they drew on established diagnostic criteria, the process was frequently complicated by systemic barriers, including long waiting times, short blocks of intervention, limited access to multidisciplinary teams, and the additional interpretive challenges presented by multilingualism and language exposure.

Compounding these structural factors was a pervasive sense of clinical tentativeness. While participants expressed strong familiarity with diagnostic criteria, they often described a hesitation in "owning" the diagnosis, particularly in the absence of professional support. As such, the diagnosis of DLD was frequently framed as a "working" label; one that offered utility but also implied ongoing doubt.

This theme is presented through two interrelated subthemes: the first attends to the structural and systemic conditions that shape the diagnostic process, while the second considers how clinicians experience and respond to uncertainty in their own diagnostic decision-making.

1.1. Structural and systemic barriers. Participants emphasised that DLD diagnosis is very much a process rather than a singular moment of decision, and all clinical participants described the importance of taking a longitudinal view on Response-to-Intervention (RtI). However, clinicians were unanimous that limited service structures, such as restricted therapy blocks and long waiting lists, prevented a timely diagnosis. Additionally, a change of staff was seen as disrupting the flow of the diagnostic process.

> *" …we need to see them get through some intervention so that we know what kind of progress they've made. Which means that, that's delayed, at least by, you know, them coming back in for another round of intervention, which is, you know, a year and a half, or potentially more, depending on what's happening at the moment with services"* (FG-C3)

In this context, gathering pertinent information from sources external to clinical settings was deemed essential but was seen as challenging.

> *"…it's quite hard to contact schools … and get that broader view"* (FG-C2)

The DLD diagnosis is often made after "ruling out" other "disorders" (i.e., autism, learning difficulties) and may be proposed as a "working diagnosis" for some time. One challenge noted by clinicians here was access to multidisciplinary assessments which would support differentiation of DLD from other neurodevelopmental differences.

> *"… even psychologists would be hemming along between ASD and DLD and you'll see that written, in psychological reports and recommend 'A review in the future' to kind of tease that out again"* (FG-C1)

In an increasingly multilingual population, clinicians reported additional complexity in distinguishing between language exposure effects and persistent language difficulties characteristic of DLD. This requires an additional layer of exploration with families and schools to diagnose.

> *"what's an exposure issue, and what is a genuine disorder" (FG-C3)*

1.2. Degree of clinical confidence. Despite clinicians having a good understanding of the criteria necessary in diagnosis there was a lack of clinical confidence in giving a diagnosis.

> *"I do think the diagnosis is super important … we're not just there yet, in terms of being confident about delivering it"* (FG-C2)

Limitations within individual clinicians' confidence in giving a diagnosis of DLD was associated with a lack of confidence in unilateral diagnoses. Several clinicians noted that diagnosing without the support of a multidisciplinary team placed considerable responsibility on individual practitioners, contributing to their uncertainty.

"… very reassuring to hear from somebody else..., It's okay to say that this is a working diagnosis" (FG-C1)

Familiarity with diagnostic profiles and observational skills used to measure Response-to-Intervention (RtI) acquired over the years results in a greater certainty around distinguishing diagnosis. Clinicians shared that the breadth of experience means greater confidence.

"it's not outside of the scope of anyone's role. The term has evolved and the criteria has evolved a little bit. But it's what we have always been doing, identifying children who have a different learning mechanism for language" (FG-C3)

Lack of confidence occurs where there is a sense that the profile is complex. This tentativeness was due to a desire to be sure, underpinned by the recognition that giving a diagnostic label is a serious undertaking with social and emotional impact for children and families.

"… because that's the worst fear, like, and I say, fear in research, but that happens, how come a child goes in with one diagnosis and then …" (FG-C4)

Continuing Professional Development opportunities were noted as having an important role in growing confidence to 'diagnose' DLD. Voluntary participation in entities such as the DLD SIG was valued. However, clinicians commented that the initial flurry of information following the CATALISE consensus paper was less accessible over recent years. A welcome positive support is the increased information available to share from online sources.

"[I] feel a lot more confident handing a leaflet to a parent and saying, 'This is what I think your child has, these are the sorts of things that are making me think that." (FG-C1)

While Theme 1 explored the structural and professional challenges involved in reaching a diagnosis of DLD, participants also emphasised the importance (and the difficulty) of what follows: communicating that diagnosis in a way that supports understanding and acceptance. For both clinicians and parents, the act of conveying the meaning and implications of DLD emerged as a further site of complexity. Theme 2 explores this communication process, focusing on how clinicians explain the diagnosis to parents, how parents make sense of it, and the varied ways in which the diagnosis is, or is not, shared with children.

2. Communicating a DLD diagnosis

Communicating a diagnosis of DLD involves more than simply naming a condition; it is a layered process shaped by parents' existing knowledge, clinicians' communication approaches, and the developmental stage of the child. This theme explores the variability in how DLD is conveyed and understood by parents and children, highlighting tensions between clarity and ambiguity, reassurance and uncertainty.

2.1. Explaining DLD to parents. The communication of the meaning of a DLD diagnosis and, importantly, the implications of this diagnosis emerged as a key area of complexity within the findings. Clinicians emphasised the importance of parents having a full understanding of the impact of DLD.

"I think it's a heavy label for them to get their head around [...] to understand that, you know, it's going to be a lifelong condition" (FG-C3)

However, many parents described receiving little guidance at the point of diagnosis and felt unclear about what DLD actually meant for their child:

"… we're googling things, and we're looking at things …. There isn't really much. It's very wishy-washy (...) it's kind of a bit fluffy, DLD, you're getting this diagnosis because we're out ruled everything else. But what is it? Where are we going with it?" (FG-P3)

This was further complicated by the strong affective aspects of the diagnostic processes, with parents often admitting to mixed emotional responses. Despite this confusion, some parents framed the diagnosis as a relief, particularly when compared to concerns about other neurodevelopmental conditions.

'It was kind of relief, because, to be honest, I was expecting something worse" (FG-P2)

"that fear of autism, you know, the talk of autism, of the possibility of autism" (FG-C4)

Parents are important advocates for their children, and so it is critical that they can access the DLD diagnosis for their child in a timely way *and* with full clarity and understanding. Many parents reported that they felt they had to 'pester' clinicians for more information:

"… I found that I had to kind of ask specific questions, to find out what DLD exactly was (...) if you could sit down with them on your own, like a separate meeting to the one that you have when you go in with your child (...) perhaps an hour with you and your husband (...) they can go through all the different scenarios with you" (FG-P3)

The quality and depth of post-diagnosis support in our study varied in parent reports and may be dependent upon the approach of individual clinicians, and perhaps the practice within a particular team. Some parents felt there was a definite gap between receiving a diagnosis and having this explained to them:

"…she's just sent me the report (...) there was no follow up from her" (FG-P3)

Parents often felt responsible for initiating their own understanding and expressed a desire for structured, dedicated conversations about their child's diagnosis.

2.2. Explaining DLD to children. Despite having an unclear understanding of DLD themselves, in the majority of cases, it was the parents who were left with the responsibility of communicating about DLD to their children. In effect, parents were the default communicators to the child regarding the reality of the DLD diagnosis and of its implications. Whether or not, and how, they shared this knowledge with their children, depended on their assessment of children's level of understanding, the impact of this conversation (for example, at the child's transition to or from DLD class to mainstream school), and also parental own understanding and confidence. For example, one parent noted:

"but now, the fact that he's kind of much easier to understand, and he's in his last year in the language class, and that we just had DLD Day we do say that DLD a bit more now" (FG-P3)

Concerningly, however, these conversations did not always happen:

"we didn't really speak about it (...) not that he wouldn't understand but, like he's never asked why he had to change schools or why he's doing speech" (FG-P4)

This suggests that in some families, the diagnosis remained unspoken—either due to perceived developmental unreadiness or parental discomfort. This may have impacts for the child's identity, self-image and levels of future autonomy, as well as possible future relationship challenges between parents and children.

Clinicians noted that older children may be more included in the process, and a few clinicians noted that they would sometimes have a session with the child and invite them to attend a 'DLD and Me' group, but both clinicians and parents felt that children younger than about seven or eight years of age were too young 'to understand' it.

"The speech and language therapist would kind of talk [to the parent] in front of the child a lot about like, the diagnosis, and then also about whether or not they'd get into the language class, and they just, just found that a little bit tough' (FG-P4)

These findings point to the emotional and informational complexity of communicating a DLD diagnosis, and the level of diversity involved across the participants. Many parents felt under-informed and unsupported, often left to make sense of the diagnosis. Concerningly, they often were left to explain DLD to their child. This highlights the need for clearer, more consistent communication practices following diagnosis.

3. Utility of DLD diagnosis for children and families

This theme examines how the DLD diagnosis can act as a gateway to targeted supports and advocacy opportunities but also reveals inconsistencies in how services respond to the label. While many parents and clinicians viewed the diagnosis as a means of unlocking support, particularly access to language classes, both groups also expressed concern about the fragility of these supports. This is particularly the case at transition points or outside of designated settings.

3.1. Access to service provision and supports. Clinicians and parents universally agreed that the usefulness of a DLD diagnosis was primarily linked to the possibility of more intensive therapy and crucially, access to a small group teaching that is offered in a language class setting.

"I was very grateful that the diagnosis came just in time for her to be eligible for the language class." (FG-P3)

The experience of being in a language class was seen as important in facilitating children's understanding and acceptance of DLD, bringing a sense of reassurance and belonging. Similar findings were seen in Ireland in relation to the experience of children attending reading classes [28]. However, in Ireland, only a small proportion of children with DLD attend language classes and a DLD diagnosis does not guarantee a place in these classes. This lack of consistency of support outside of language classes was highlighted by many clinicians.

"I don't know that the schools are really treating a child with DLD in a specific way" (FG-C3)

This suggests that outside of specialist settings, the diagnostic label does not reliably translate into differentiated support in mainstream contexts—raising questions about the structural recognition of DLD in general education.

Both parents and clinicians noted stress around transition points and sudden withdrawal of support permeated parent narratives.

"Every year they try and pull resources off him every single year" (FG-P1)

Parents stressed that they continued to fight for children with DLD to be acknowledged in relation to access to various educational accommodations (for example, automatic exemption from the compulsory study of Irish).

3.2. From label to identity: building self-advocacy in children with DLD. The DLD Awareness Day was mentioned by both clinicians and parents as especially important for building confidence and a sense of "ownership" of DLD diagnosis for children who have it. Clinicians emphasised that the capacity for children and young people to understand and communicate their own needs was not only a therapeutic goal, but a strategic response to systemic limitations in service provision. As one participant explained:

"… ultimately, we're going to be able to give them very limited amounts of therapy" (FG-C2)

In this context, supporting children (particularly adolescents) to develop confidence in self-advocacy was seen as an essential therapeutic outcome. This was not presented as a late-stage add-on but as a long-term approach embedded from early stages of intervention.

"So, from as young an age as we can, we would start that, and when they get up into the teenage years then, it's more, a lot more focused on that than it would be impairment focused therapy" (FG-C2)

Clinicians described situations in which children with DLD had experienced mental health difficulties or poor school engagement due to a lack of understanding around their communication needs. The diagnostic conversation, in these cases, was reframed as an opportunity to build understanding and agency.

"… I did spend quite a bit of my first session kind of talking through it with her and with her mum and, kind of talking through almost like advocacy about how she could advocate for herself. How she could, you know, talk to her teachers about this, things like that" (FG-C1)

These accounts suggest that, when embedded meaningfully, a DLD diagnosis can support more than access to services. It can enable children to participate more fully in their own learning and social environments by giving language to their experiences and strategies to navigate them, thus supporting autonomy and agency across the developmental trajectory.

4. Going forward and recommendations

This theme explores how participants viewed the visibility of DLD across health, education, and public settings. While some gains were noted, particularly through initiatives such as DLD Awareness Day, participants described an overall lack of recognition at national and institutional levels. Both clinicians and parents expressed concern that responsibility for building awareness was falling disproportionately on families, who themselves were often still trying to understand the diagnosis.

4.1. Systemic gaps in DLD awareness and recognition. All participants emphasised the importance of fostering greater awareness of DLD among other healthcare professionals, in the education sector, community and the wider society. Participants described DLD as underrepresented in public discourse, lacking the policy presence or advocacy infrastructure seen in other neurodevelopmental conditions. Both groups noted the DLD Awareness Day had positive impacts on awareness and on support. Efforts to raise awareness were noted to be critical, with one clinician noting:

"… not just the people who were attending kind of from the community, but actually, our own kind of colleagues in healthcare" (FG-C1)

However, while initiatives like DLD Awareness Day were valued, they were insufficient in themselves, and DLD was seen as needing a more national level recognition, similar to, for instance, advocacy for autism, as well as inclusion in medical training. As one parent noted:

"I'm an intellectual disability nurse, so I would be very au fait with speech and language, it would be part of my job, all my life, I used to watch and stuff, but I had never heard of DLD until he was diagnosed" (FG-P1)

4.2. Enhancing recognition in the education sector. In relation to the Education sector, both parents and clinicians stressed the necessity of teacher education and teacher professional development on DLD. This should include

embedding the knowledge on DLD in initial teacher education. Parents felt that this advocacy responsibility is currently placed on them:

"It shouldn't have to be parents that have to educate them" (FG-P2)

Considering that parents themselves lack clarity on DLD, this is not just inadequate, but also not equitable:

"Every year we have the same conversations with the teacher" (FG-P1)

Both clinicians and parents offered concrete suggestions for change, including (a) the development of educator resources, (b) formal education on DLD in initial and continuing teacher education, and (c) the creation of peer support networks. These proposals are further discussed in the implications section below.

Discussion

Our research provides first-hand evidence from parents and clinicians in Ireland in relation to their perspectives on the DLD diagnosis. This is an under-researched area both internationally [7] and in Ireland [13,29]. By hearing the voices of both the parents and the clinicians, we have the ability to identify obstacles. This is needed to support change.

Our findings highlight both systemic and individual challenges to both early and timely DLD diagnosis in Ireland. This is concerning in light of what we now know about poor prognostic factors associated with DLD [1] and negative psychosocial outcomes associated with late DLD diagnosis [30]. It seems that, in the absence of multidisciplinary assessments, clinicians are hesitant to diagnose DLD if children present with 'complex' profiles. Given the overlap in neurodevelopmental conditions [31], this is hugely problematic. Our research documented clinicians' fear of getting the "right" condition for diagnosis, which may be firstly at odds with the individual profiles of children, and secondly, acts as a barrier in accessing the needed support.

The findings of our research show that parents themselves do not have a clear understanding of DLD diagnosis. Yet it is parents (or guardians) who have a primary legal responsibility of caring for and making decisions about the child [32]. The clinical practice of communicating a DLD diagnosis to families gains particular importance here, as it influences whether or not the parents can fulfil their legal obligations. The limited understanding of DLD among parents in our study is in line with much of what has been known about DLD for some time now, namely the 'hidden' nature of DLD (and language difficulties in general) [3], as well as limited awareness of DLD in society in general [29]. Research has also shown that an improved understanding of a diagnosis reduces parental stress and increases their perceived ability to cope with their child's difficulties [33]. Thus, our research underscores the importance of not just timely, but also *comprehensible* diagnosis and support.

To promote this understanding among families, including young children, some of the parent participants have suggested a separate (not clinical) family session that would focus on explaining DLD. In their proposal of practical strategies supporting therapists in collaborating with parents, Klatte et al. [34] included an overarching strategy of 'invest time in optimising the collaboration with parents'. While seemingly obvious, this may be the first practical step that is required in the context of communicating DLD diagnosis in Ireland. We need to be able to consider feasible solutions in the context of the 'rushed' global healthcare system, with an additional barrier of very high level of staff turnover, which does not facilitate individualised and *holistic* engagement with each parent and child. Working environments were widely viewed by the clinicians as presenting barriers to collaboration with both families and other professionals.

In Ireland, demonstrating the ability to provide clients and carers with information in appropriate formats to facilitate decision-making and informed consent is among key clinical competencies, shaped during undergraduate SLT education [35]. Our research found that firstly, access to an educational pathway, namely the language class, often influences

whether or not, as well as "when" and "how", the information about DLD is shared with families. This teleological nature of DLD diagnosis in Ireland is problematic. Secondly, we found that there is an urgent need for clinical tools supporting that the information shared with families is clear, accessible, and tailored to their individual needs, including very young children who have a right to express their views on all matters that affect them and for these views to be given due weight [32,36].

The usefulness of the DLD label for families was intrinsically linked to the lack of awareness in society and especially in schools. Many parents reported their "exhaustion" from constantly explaining themselves. This is not helped by the current lack of DLD awareness in the wider society, including in initial and continuing teacher education and education policy documents in Ireland. Parents in our study were also concerned about the lack of representation of adults with DLD in DLD advocacy efforts, echoing the findings by Burnley et al. [30]. It is anticipated that with increased research, people with DLD will become more 'visible' and with timely and comprehensible diagnosis will be better equipped to advocate for themselves.

Implications

The study has important implications for a range of contexts and stakeholders. Firstly, we argue that, following DLD diagnosis, more time needs to be spent in clinical *and* non-clinical conversations with parents and children, allowing them time to process the information and to seek clarifications. There is a need to develop and promote creative tools and resources that would assist both clinicians and especially parents to explain DLD to children in a way that is accessible to all children, including very young children. Both clinicians and parents emphasized the importance of continued efforts to raise awareness and enhance knowledge of DLD, particularly in schools, as well as in the professional education of health, psychology, and education professionals. These efforts have been in progress since the CATALISE study [1] and were also highlighted by researchers in Ireland [29], and must continue.

Our findings show that parents feel isolated and unsupported in relation to both understanding and supporting this diagnosis, and that they perceive the information about DLD vague and inadequate. This calls for more than a central repository of resources that would direct parents towards effective support mechanisms (which has been established under Raising Awareness of Developmental Language Disorder national and international campaigns), but also for a promotion of parent support networks and parent advocacy groups.

Most importantly, given the reported lack of clinicians' confidence regarding DLD diagnosis, we advocate for increased access to professional development for clinicians, that is a) regularly updated in line with changing diagnostic descriptors and practice guidelines, b) clarifies the information regarding co-occurrence of DLD and other neurodevelopmental differences, and c) is based on effective models of professional learning which promote collaborative inquiry and problem solving to support sharing of expertise and clinical confidence in practice.

Limitations

While our research cannot be viewed as representative of the views of Irish clinicians and parents, in relation to our key findings, we did find a consensus among the participants both within and across focus groups. However, we do acknowledge that a dominant narrative can impact group discussion [37], thus, our interpretation of a consensus within focus groups may not be accurate. We followed the COREQ reporting guidelines [20], however, we acknowledge that the transferability of our findings are limited without including children's voices in the research.

Conclusion

Our research highlights systemic and professional challenges contributing to delays and hesitancy in diagnosing Developmental Language Disorder (DLD) in Ireland. It shows that parents frequently experience confusion and isolation due

to limited information and support. The findings underscore the need for improved clinical communication practices, increased public and professional awareness, and stronger support structures for both parents and clinicians. Both timely and comprehensible DLD diagnosis is essential to ensure that families are empowered to support their children effectively and that children with DLD receive the necessary support. Most importantly, in light of growing awareness of the co-occurrence of neurodevelopmental differences, we call for urgent, enhanced support for clinicians to build their confidence in navigating the evolving diagnostic criteria of DLD.

Supporting information

S1 Appendix. Informed consent form.
(DOCX)

S2 Appendix. Focus group schedule.
(DOCX)

Acknowledgments

We would like to thank the parents and clinicians who took part in the study.

Author contributions

Conceptualization: Sylwia Kazmierczak-Murray, Neil Kenny, Suzanne Carolan.

Data curation: Alison Doyle.

Formal analysis: Sylwia Kazmierczak-Murray, Neil Kenny, Suzanne Carolan, Alison Doyle.

Funding acquisition: Neil Kenny.

Investigation: Sylwia Kazmierczak-Murray, Neil Kenny, Suzanne Carolan.

Methodology: Sylwia Kazmierczak-Murray, Neil Kenny.

Writing – original draft: Sylwia Kazmierczak-Murray.

Writing – review & editing: Neil Kenny, Suzanne Carolan, Alison Doyle.

References

1. Bishop DVM, Snowling MJ, Thompson PA, Greenhalgh T, CATALISE consortium. CATALISE: A Multinational and Multidisciplinary Delphi Consensus Study. Identifying Language Impairments in Children. PLoS One. 2016;11(7):e0158753. https://doi.org/10.1371/journal.pone.0158753 PMID: 27392128
2. McLeod S, McKinnon DH. Prevalence of communication disorders compared with other learning needs in 14,500 primary and secondary school students. Int J Lang Commun Disord. 2007;42 Suppl 1:37–59. https://doi.org/10.1080/13682820601173262 PMID: 17454236
3. McGregor KK. How We Fail Children With Developmental Language Disorder. Lang Speech Hear Serv Sch. 2020;51(4):981–92. https://doi.org/10.1044/2020_LSHSS-20-00003 PMID: 32755505
4. Ziegenfusz S, Paynter J, Flückiger B, Westerveld MF. A systematic review of the academic achievement of primary and secondary school-aged students with developmental language disorder. Autism Dev Lang Impair. 2022;7:23969415221099397. https://doi.org/10.1177/23969415221099397 PMID: 36382072
5. Leitão S, St Clair MC, Botting N, Gibson J, Jackson E. "They don't realise how hard he has to try every day": The rewards and challenges of parenting a child with developmental language disorder. Int J Lang Commun Disord. 2025;60(2):e70003. https://doi.org/10.1111/1460-6984.70003 PMID: 39970424
6. Wilmot A, Boyes M, Sievers R, Leitão S, Norbury C. Impact of developmental language disorders on mental health and well-being across the lifespan: a qualitative study including the perspectives of UK adults with DLD and Australian speech-language therapists. BMJ Open. 2024;14(10):e087532. https://doi.org/10.1136/bmjopen-2024-087532 PMID: 39438095
7. Kulkarni AA, Chadd KE, Lambert SB, Earl G, Longhurst LM, McKean C, et al. Editorial Perspective: Speaking up for developmental language disorder - the top 10 priorities for research. J Child Psychol Psychiatry. 2022;63(8):957–60. https://doi.org/10.1111/jcpp.13592 PMID: 35365906

8. Tighe JM, Namazi M. SPICES: Disclosure practices to help caregivers digest a diagnosis of developmental language disorder. Am J Speech Lang Pathol. 2022;31(5):1919–32. https://doi.org/10.1044/2022_AJSLP-21-00295 PMID: 36007195
9. Bishop DVM. Ten questions about terminology for children with unexplained language problems. Int J Lang Commun Disord. 2014;49(4):381–415. https://doi.org/10.1111/1460-6984.12101 PMID: 25142090
10. Wright E. Special education needs provision in the real world. Int J Lang Commun Disord. 2014;49(4):448–51.
11. Ebbels S. Introducing the SLI debate. Int J Lang Commun Disord. 2014;49(4):377–80. https://doi.org/10.1111/1460-6984.12119 PMID: 25142089
12. Elliott JG, Grigorenko EL. The Dyslexia Debate. Cambridge: Cambridge University Press. 2014. https://doi.org/10.1017/cbo9781139017824
13. Gallagher AL, Finn S, Dolan R, Dunphy E. Exploring the uptake of CATALISE recommendations from the perspective of speech and language therapists working in the Irish context: A qualitative online survey. Adv Commun Swallowing. 2023;26(1):13–23.
14. Ash AC, Christopulos TT, Redmond SM. "Tell Me About Your Child": A Grounded Theory Study of Mothers' Understanding of Language Disorder. Am J Speech Lang Pathol. 2020;29(2):819–40. https://doi.org/10.1044/2020_AJSLP-19-00064 PMID: 32348158
15. Porter KL, Oetting JB, Pecchioni L. Caregivers' Perceptions of Speech-Language Pathologist Talk About Child Language and Literacy Disorders. Am J Speech Lang Pathol. 2020;29(4):2049–67. https://doi.org/10.1044/2020_AJSLP-20-00049 PMID: 32857589
16. Jacobs D, Steyaert J, Dierickx K, Hens K. Parents' views and experiences of the autism spectrum disorder diagnosis of their young child: a longitudinal interview study. Eur Child Adolesc Psychiatry. 2020;29(8):1143–54. https://doi.org/10.1007/s00787-019-01431-4 PMID: 31686239
17. Almog N, Kassel O, Levy N, Gabai T. Mapping the Dilemmas Parents Face with Disclosing Autism Diagnosis to their Child. J Autism Dev Disord. 2023;53(10):4060–75. https://doi.org/10.1007/s10803-022-05711-y PMID: 35951204
18. Thomas S, Schulz J, Ryder N. Assessment and diagnosis of Developmental Language Disorder: The experiences of speech and language therapists. Autism Dev Lang Impairments. 2019;4. https://doi.org/10.1177/2396941519842812
19. Roulstone SE, Lindsay G. The perspectives of children and young people who have speech, language and communication needs, and their parents. 2012. https://www.education.gov.uk/publications/standard/publicationDetail/Page1/DFE-RR247-BCRP7
20. Tong A, Sainsbury P, Craig J. Consolidated criteria for reporting qualitative research (COREQ): a 32-item checklist for interviews and focus groups. Int J Qual Health Care. 2007;19(6):349–57. https://doi.org/10.1093/intqhc/mzm042 PMID: 17872937
21. Thomas S, Schulz J, Ryder N. Assessment and diagnosis of Developmental Language Disorder: The experiences of speech and language therapists. Autism Dev Lang Impair. 2019;4. https://doi.org/10.1177/2396941519842812
22. Teixeira E, Silva C, Vicente A. Development and validation of an interview guide for examining the effects of sports careers on the quality of life of retired Portuguese football players. Front Psychol. 2024;15:1374784. https://doi.org/10.3389/fpsyg.2024.1374784 PMID: 38533209
23. Turner D III. Qualitative Interview Design: A Practical Guide for Novice Investigators. TQR. 2014. https://doi.org/10.46743/2160-3715/2010.1178
24. Squires A. Methodological challenges in cross-language qualitative research: a research review. Int J Nurs Stud. 2009;46(2):277–87. https://doi.org/10.1016/j.ijnurstu.2008.08.006 PMID: 18789799
25. van Nes F, Abma T, Jonsson H, Deeg D. Language differences in qualitative research: is meaning lost in translation?. Eur J Ageing. 2010;7(4):313–6. https://doi.org/10.1007/s10433-010-0168-y PMID: 21212820
26. Temple B, Young A. Qualitative Research and Translation Dilemmas. Qualitative Res. 2004;4(2):161–78. https://doi.org/10.1177/1468794104044430
27. Bingham AJ. From Data Management to Actionable Findings: A Five-Phase Process of Qualitative Data Analysis. International J Qualitat Methods. 2023;22. https://doi.org/10.1177/16094069231183620
28. Casserly AM. The socio-emotional needs of children with dyslexia in different educational settings in Ireland. Res Spec Educ Needs. 2012;13(1):79–91. https://doi.org/10.1111/j.1471-3802.2011.01227.x
29. Gibbons M, Coughlan K, Gallagher A. Hidden in plain sight: A qualitative exploration of teachers and children's perspectives on supporting developmental language disorder in school. ACS. 2023;26(1):3–12. https://doi.org/10.3233/acs-220010
30. Burnley A, St Clair M, Dack C, Thompson H, Wren Y. Exploring the Psychosocial Experiences of Individuals with Developmental Language Disorder During Childhood: A Qualitative Investigation. J Autism Dev Disord. 2024;54(8):3008–27. https://doi.org/10.1007/s10803-023-05946-3 PMID: 37338728
31. Embracing Complexity. Embracing complexity in diagnosis: Multi-diagnostic pathways for neurodevelopmental conditions. London: Embracing Complexity; 2019. Available from: https://embracingcomplexity.org.uk/reports#ecdiagnosis
32. United Nations General Assembly. Convention on the Rights of the Child. New York: United Nations. 1989.
33. Creen J, Kennedy-Behr A, Donkin R, Verdonck M. Understanding parent engagement in advanced allied health single session therapy for children with developmental and behavioural disabilities. Child Care Health Dev. 2024;50(1):e13148. https://doi.org/10.1111/cch.13148 PMID: 37369641
34. Klatte IS, Ketelaar M, de Groot A, Bloemen M, Gerrits E. Collaboration: How does it work according to therapists and parents of young children? A systematic review. Child Care Health Dev. 2024;50(1):e13167. https://doi.org/10.1111/cch.13167 PMID: 37724049

35. National Clinical Competency Framework. National Student Clinical Competencies Performance Indicators. 2015.

36. Kazmierczak-Murray S, O'Mahony K, Carey A. A literature review on the inclusion of disabled children and young people in participation in decision-making. Department of Children, Equality, Disability, Integration and Youth. 2024. https://hubnanog.ie/a-literature-review-on-the-inclusion-of-disabled-children-and-young-people-in-participation-in-decision-making/

37. Flynn R, Albrecht L, Scott SD. Two Approaches to Focus Group Data Collection for Qualitative Health Research. Int J Qualitative Methods. 2018;17(1). https://doi.org/10.1177/1609406917750781
